# HIV care and treatment factors associated with improved survival during TB treatment in Thailand: an observational study

**DOI:** 10.1186/1471-2334-9-42

**Published:** 2009-04-13

**Authors:** Jay K Varma, Sriprapa Nateniyom, Somsak Akksilp, Wiroj Mankatittham, Chawin Sirinak, Wanchai Sattayawuthipong, Channawong Burapat, Wanitchaya Kittikraisak, Patama Monkongdee, Kevin P Cain, Charles D Wells, Jordan W Tappero

**Affiliations:** 1 U.S. Centers for Disease Control and Prevention, Atlanta, USA; 2 Thailand Ministry of Public Health – U.S. CDC Collaboration, Nonthaburi, Thailand; 3 Thailand Ministry of Public Health, Nonthaburi, Thailand; 4 Office of Disease Prevention and Control 7, Ubon Ratchathani, Thailand; 5 Bamrasnaradura Institute, Nonthaburi, Thailand; 6 Bangkok Metropolitan Administration, Bangkok, Thailand; 7 Phuket Provincial Health Office, Phuket, Thailand

## Abstract

**Background:**

In Southeast Asia, HIV-infected patients frequently die during TB treatment. Many physicians are reluctant to treat HIV-infected TB patients with anti-retroviral therapy (ART) and have questions about the added value of opportunistic infection prophylaxis to ART, the optimum ART regimen, and the benefit of initiating ART early during TB treatment.

**Methods:**

We conducted a multi-center observational study of HIV-infected patients newly diagnosed with TB in Thailand. Clinical data was collected from the beginning to the end of TB treatment. We conducted multivariable proportional hazards analysis to identify factors associated with death.

**Results:**

Of 667 HIV-infected TB patients enrolled, 450 (68%) were smear and/or culture positive. Death during TB treatment occurred in 112 (17%). In proportional hazards analysis, factors strongly associated with reduced risk of death were ART use (Hazard Ratio [HR] 0.16; 95% confidence interval [CI] 0.07–0.36), fluconazole use (HR 0.34; CI 0.18–0.64), and co-trimoxazole use (HR 0.41; CI 0.20–0.83). Among 126 patients that initiated ART after TB diagnosis, the risk of death increased the longer that ART was delayed during TB treatment. Efavirenz- and nevirapine-containing ART regimens were associated with similar rates of adverse events and death.

**Conclusion:**

Among HIV-infected patients living in Thailand, the single most important determinant of survival during TB treatment was use of ART. Controlled clinical trials are needed to confirm our findings that early ART initiation improves survival and that the choice of non-nucleoside reverse transcriptase inhibitor does not.

## Background

Globally, an estimated nine million new cases of TB occur annually, resulting in almost two million deaths [1]. The HIV pandemic has increased TB incidence and mortality, because HIV greatly increases the risk of developing TB disease and complicates diagnosis and treatment of TB [2]. In Southeast Asia, mortality from HIV-associated TB is particularly high; after diagnosis of TB, up to 50% of HIV-infected patients may die during TB treatment [3,4]. Prospective epidemiologic studies from resource rich countries and Thailand have shown that anti-retroviral therapy (ART) greatly reduces mortality during TB treatment [5-9]. In patients not prescribed ART, co-trimoxazole has been shown to reduce mortality [10,11]. The World Health Organization (WHO) currently recommends that all HIV-infected TB patients receive co-trimoxazole and that patients with clinical evidence of AIDS or CD4+ T-lymphocyte counts (CD4) <350 cells/μL initiate ART during TB treatment [2]. Despite international recommendations and the proven benefit of ART, physicians remain reluctant to prescribe ART to HIV-infected TB patients, because of concerns about overlapping toxicity, drug-drug interactions, pill burden, and immune reconstitution inflammatory syndrome (IRIS) [12]. Major research questions also remain unanswered, including the optimum time to initiate ART, the optimum ART regimen in HIV-infected TB patients, and the added value to ART of co-trimoxazole and other opportunistic infection prophylaxis medications in reducing mortality during TB treatment [13]. Clinical trials are currently in progress to help answer some of these questions [14].

Thailand has been greatly affected by the TB/HIV syndemic: TB develops annually in 91,000 persons, 15–20% of whom are HIV-infected [1,15,16]. While awaiting the results of clinical trials in other countries, we sought to evaluate factors associated with mortality in a prospective, multi-center, observational study of HIV-infected patients treated for TB in Thailand. Our specific purpose was to identify and quantify the relative benefit of biomedical interventions associated with survival during TB treatment, to evaluate the optimum time to initiate ART, and to evaluate adverse events and survival associated with different ART regimens.

## Methods

### Setting

Patients were recruited from 32 public TB treatment facilities from May 2005 through September 2006. The catchment area included the entire provinces of Ubon Ratchathani and Phuket, 10 districts in Bangkok, and the national infectious diseases hospital in Nonthaburi province. All facilities were government-supported hospitals that had both outpatient TB clinics and inpatient treatment centers from which patients could be enrolled. Excluding the national hospital, which has no base population, the population covered was 3.1 million persons. In all facilities, TB patients are routinely registered and treated in a specialized clinic; physicians in these facilities are instructed, but not required, to use national guidelines for TB diagnosis and treatment. As part of a multi-site demonstration project known as the Thailand TB Active Surveillance Network, public health staff in these facilities recorded standardized data about all patients diagnosed with TB, and clinical staff were encouraged to send sputum specimens for mycobacterial culture and to provide HIV counseling and testing [17].

### Enrollment

Patients who were registered in the Thailand TB Active Surveillance Network and who were documented to be HIV-infected were screened for eligibility by study staff. HIV-infected TB patients were eligible for the study if they had been receiving anti-TB treatment for less than four weeks at the time of enrollment and were greater than 17 years old, not known to be pregnant, and not incarcerated. Additionally, patients had to report that they planned to remain in the study sites' TB treatment program for the duration of TB treatment. Eligible patients were informed about the study and, if they agreed to participate, were consented for participation.

### Study evaluations

At enrollment, trained clinical nurses interviewed patients using a standardized questionnaire that asked about a large number of demographic, knowledge, behavioral, and medical characteristics. Clinic physicians recorded physical examination findings on study forms. Blood and sputum specimens were collected for testing. Patients initiated treatment for TB, HIV, and other diseases according to physician preference; no efforts were made to modify routine clinical practice.

After two months of initial TB treatment and at the end of TB treatment, patients were interviewed and examined again. Study staff abstracted medical records of patients who received care between study visits. Patients were not followed after TB treatment ended. To determine if patients died after defaulting, we linked patients who defaulted during TB treatment to the Thai government's vital status registry, a system known to be relatively complete [18]. Patients who died within three months of defaulting were classified as deaths during study follow-up. In total, 11 patients who were originally classified as defaulters were later re-classified as deaths.

### Laboratory studies

Patients diagnosed with pulmonary TB provided sputum specimens for smear microscopy. Unprocessed sputum was analyzed in TB clinics using the Ziehl-Neelsen acid-fast stain method. Similarly, as part of our Active Surveillance Network program, we encouraged, but did not require, that at least one sputum specimen be sent for mycobacterial culture, identification, and drug-susceptibility testing. Mycobacterial culture was performed at one laboratory in each province using both Lowenstein-Jensen and mycobacterial growth indicator tube (MGIT) media, according to standard methods [19]. Identification and first-line drug-susceptibility testing was performed at reference laboratories in Bangkok; indirect drug susceptibility testing using the proportion method was performed on BACTEC MGIT 960 [20].

Blood specimens collected at enrollment underwent a complete blood count, testing for hepatitis B surface antigen (HBsAg) and hepatitis C antibody (anti-HCV), enumeration of CD4 using a flow cytometer, and measurement of serum albumin, creatinine, aspartate aminotransferase (AST), alanine aminotransferase (ALT), and total bilirubin. Plasma specimens from selected patients were tested for HIV RNA using polymerase chain reaction (Amplicor HIV Monitor Test, version 1.5, Roche Molecular Systems, Branchburg, NJ, USA).

### Definitions

Patients were defined as having bacteriologic-confirmed TB if at least one specimen collected at any time before or during treatment was positive for acid-fast bacilli and/or culture-positive for *Mycobacterium tuberculosis *(MTB). Patients with specimens that grew non-tuberculous mycobacteria and not MTB were considered not to have TB and excluded from all analyses.

We developed composite variables to measure factors initially hypothesized to be associated with death during TB treatment. Patients were considered to have a delay in seeking TB care if they reported: (a) having a cough lasting greater than one month before TB diagnosis; or (b) having other symptoms that lasted longer than 14 days and self-assessed these symptoms as being severe. Patients were considered to have a delay in HIV diagnosis if their CD4 count at the time of HIV diagnosis was <200 cells/μL; we used standard assumptions to estimate CD4 count at the time of HIV diagnosis for patients with a pre-existing HIV diagnosis, no CD4 count recorded at the time of HIV diagnosis, and no history of ART treatment [21-23].

Except for peripheral lymphatic TB, patients were considered to have severe TB disease if they had extra-pulmonary TB or had pulmonary TB with any of the following characteristics: self-reported weight loss, hemoptysis, dyspnea, or chest x-ray findings of a cavity or infiltrates involving >1/3 of either lung. Patients were considered to have severe HIV disease if their CD4 count at study enrollment was <200 cells/μL. Patients were considered to be receiving inadequate TB treatment if the anti-TB regimen prescribed was not consistent with international recommendations or was inadequate because of documented MTB drug resistance. Patients were considered to have elevated liver-associated enzymes if they had an AST ≥ 120 mEq/L, ALT ≥ 165 mEq/L, or total bilirubin >2 mg/dL.

We defined IRIS as worsening fever, cough, shortness of breath, lymphadenopathy, radiographic abnormalities, or extra-pulmonary TB lesions in a patient that was being treated with ART, had initially improved clinically after initiating TB treatment, and had no other diagnosis that could explain their new symptoms [24,25].

### Analytical methods

We calculated medians and proportions to describe characteristics of our study population. To determine risk factors for death, we restricted our analysis to patients who were never previously treated with TB and who were known to be either alive or dead at the end of TB treatment. We calculated time from TB treatment initiation to death or to the end of TB treatment. We created an "unknown" category for any categorical covariate with missing data for ≥10 observations. After confirming that the proportional hazards assumption was met, we constructed a Cox proportional hazards model that included factors hypothesized to be associated with death and factors that were consistently associated with death at p ≤ 0.20 in bivariate analysis of all patients and of bacteriologic-confirmed patients. For this analysis, we excluded patients who started co-trimoxazole, fluconazole and ART before TB treatment initiation, because we could not account for whether they had been continuously taking these medicines. Two-way interaction terms were generated as products of covariates and entered in the models; none were retained in the final models. We also constructed Kaplan-Meier survival curves adjusted for factors associated with mortality to evaluate the association between ART and time to death.

To measure the association between timing of ART initiation and mortality during TB treatment, we restricted our proportional hazards analysis to patients that initiated ART during TB treatment. Because our sample size for this analysis was small, we only adjusted for selected factors, such as TB and HIV disease severity. We excluded 10 patients who were treated with a non-standard or ineffective anti-TB drug regimen from this analysis, because the small number made our model unstable.

To determine whether the choice of ART regimen was associated with different TB treatment outcomes, we analyzed the likelihood of treatment success (cured or completed TB outcomes) vs. a composite end point of death and default or death only for patients that received nevirapine-containing regimens versus those that received efavirenz-containing regimens.

### Ethical review

This study was approved by the ethical review committees at the U.S. Centers for Disease Control and Prevention, the Thailand Ministry of Public Health, and the Bangkok Metropolitan Administration.

## Results

### Enrollment

**Figure 1:**
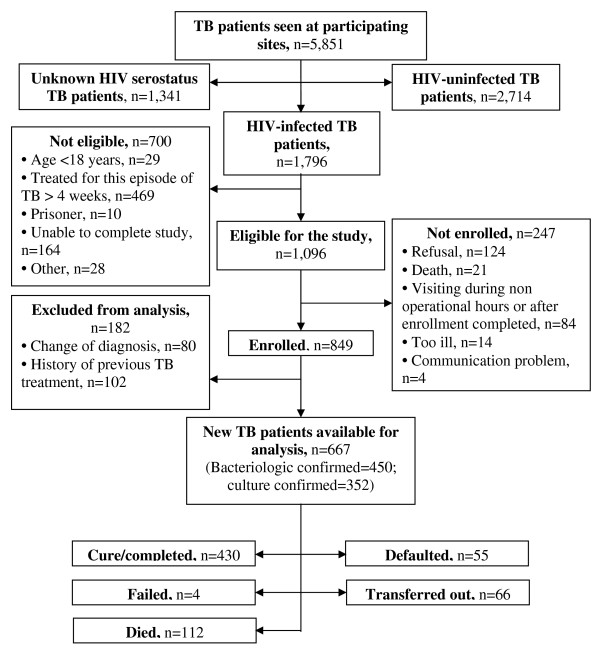
**Enrollment of HIV-infected tuberculosis patients in study**.

From May 2005 – September 2006, 5,851 TB patients were registered for treatment in the participating sites, and 1,796 (31%) were known to be HIV-infected. [Figure 1] Among the 700 patients not eligible for the study, the most common reason for ineligibility was patients taking anti-TB treatment for at least four weeks before presenting to a participating facility (n = 469). Of the 1,096 eligible patients, 849 (78%) were enrolled. We later excluded from analysis patients who reported previously being treated for TB or subsequently were diagnosed as not having TB. Of the 80 patients excluded because of a change in diagnosis, 70 (87%) had non-tuberculous mycobacteria diagnosed. The remaining 667 HIV-infected, new TB patients were eligible for analysis.

### Characteristics of cases

Most patients were men with a median age of 34 years (range, 18–77).[Additional file 1, **Table 1**] Pulmonary TB was diagnosed in 387 (58%), extra-pulmonary TB in 206 (31%), and both pulmonary and extra-pulmonary TB in 74 (11%). Bacteriologic-confirmation of TB was documented in 450 (68%), 352 (78%) of whom had culture-confirmed MTB. Resistance to isoniazid and rifampin (or multi-drug resistant [MDR] TB) was diagnosed in 16 (2%). Over 80% had CD4 < 200 cells/μL; among 12 patients with CD4 > 200 cells/μL who had HIV RNA viral load test performed, 7 (58%) had greater than 100,000 copies/mL. Serologic testing for viral hepatitis identified 54 (8%) positive for HBsAg and 192 (30%) for anti-HCV. At the beginning of TB treatment, 110 (17%) had elevated liver-associated enzymes.

### TB and HIV care and treatment

A health care worker directly observed TB treatment (DOT) for 191 (29%) patients. Of all 667 patients, 430 (65%) were cured or completed TB treatment; 112 (17%) died during TB treatment.

Rates of opportunistic infection prophylaxis use were high: before TB treatment, 215 (32%) were taking co-trimoxazole and 117 (18%) fluconazole; during TB treatment, an additional 338 (51%) began using co-trimoxazole and 259 (39%) fluconazole.

ART was prescribed to 273 (41%) patients during TB treatment, 73 (27%) of whom were receiving ART before TB diagnosis. In the 200 patients that initiated ART at the beginning or during TB treatment, the median time between TB diagnosis and ART initiation was 62 days (range, 0–386 days). Among all 273 patients receiving ART, 113 (41%) took an efavirenz-containing regimen, and 147 (54%) took a nevirapine-containing regimen. Of the 147 patients who took nevirapine, 5 (3%) switched to efavirenz during TB treatment; of these, two switched regimens at 29 and 242 days into TB treatment, respectively, while the exact date of medication change was not recorded for other three patients. For later analyses, all five patients were classified as taking a nevirapine-containing regimen. The remainder took other ART regimens (n = 3) or had incomplete data about the drugs or dates involving their ART regimen (n = 10).

### Risk factors for death during TB treatment

**Figure 2:**
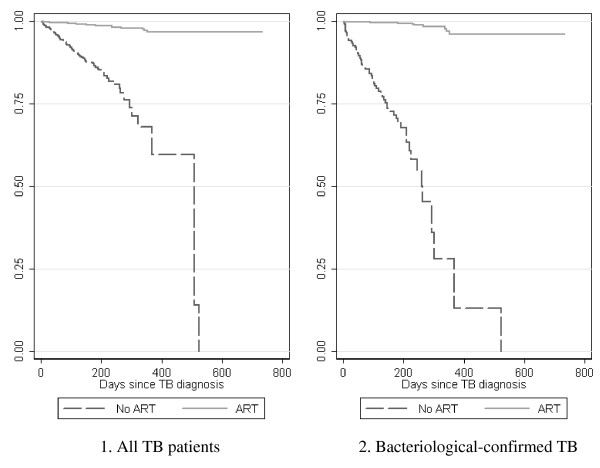
**Cumulative survival probability for HIV-infected TB patients that received or did not receive ART**. TB, tuberculosis; HIV, human immunodeficiency virus; ART, anti-retroviral therapy. Kaplan-Meier survival curves were adjusted for covariates associated with mortality.

Unadjusted mortality rates, stratified by microbiologic status and various patient characteristics, are shown in Additional file 1, **Table 2**. In proportional hazards analysis, co-trimoxazole, fluconazole, and ART use were associated with a lower risk of death in all TB patients. In the subset of bacteriologic-confirmed patients, fluconazole and ART use remained strong protective factors.[Additional file 1, **Table 3**] In the analyses of bacteriologic-confirmed patients, DOT for TB was associated with a lower risk of death; while hospitalization at enrollment, having lower CD4, and a missing albumin level were associated with higher risk of death. The survival probability for all patients and bacteriologic-confirmed patients is shown in Figure 2.

### Timing of ART

**Figure 3:**
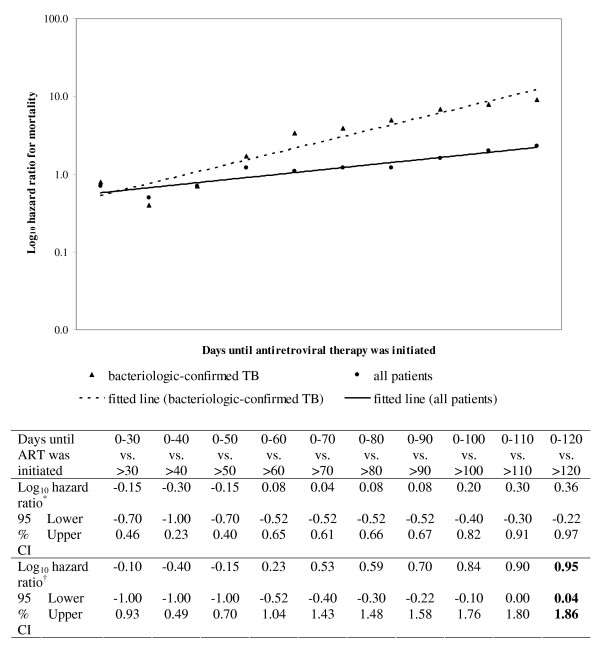
**Log hazard ratio for mortality among all HIV-infected tuberculosis patients (n = 174) and patients who had bacteriologic-confirmed tuberculosis (n = 127) and initiated ART during anti-tuberculosis treatment**. TB, tuberculosis, HIV, human immunodeficiency virus; ART, anti-retroviral therapy; HR, hazard ratio; CI, confidence interval; only those who received a TB regimen of known efficacy and started ART at the beginning or during TB treatment were included; the analyses were adjusted for TB disease severity and CD4. *Among all TB patients. ^†^Among bacteriologic-confirmed TB patients.

In a proportional hazards model adjusted for TB disease severity and CD4, the risk of death increased the longer ART was delayed. [Figure 3] The association between timing of ART and death was only statistically significant, however, when comparing those that initiated ART within the first 120 days of TB treatment to those who started later: among bacteriologic-confirmed cases, hazard ratio [HR] 9.0, 95% confidence interval [CI] 1.1–73.0.

### Adverse events and ART choice

Adverse events occurred with similar frequency in ART treated and untreated patients.[Additional file 1, **Table 4**] Rash was reported more frequently in ART-untreated patients (18%) than in patients receiving either nevirapine- (12%) or efavirenz-containing (11%) regimens, but this was not statistically significant (p-value = 0.09). Fourteen patients (2% of all; 5% of those taking ART) met our case definition of IRIS; IRIS occurred in 7 (5%) patients taking nevirapine regimens and 7 (6%) taking efavirenz regimens.

Adjusting for factors associated with TB treatment success, we found that efavirenz- or nevirapine-containing ART was associated with a decreased risk of death or of death and default, compared with no ART, and that there was no significant difference in outcomes when comparing nevirapine- vs. efavirenz-containing regimens.[Additional file 1, **Table 5**]

## Discussion

In this prospective, multi-center observational study of HIV-infected TB patients, we found that patients who took ART had one-fifth the risk of dying than those who did not take ART, and that the risk of dying was further reduced with early ART initiation and concomitant use of fluconazole. The choice of non-nucleoside reverse transcriptase inhibitor (NNRTI) in the ART regimen did not alter the risk of adverse events or death.

Our study adds to previous observational data about the benefit of ART on survival during TB treatment, because it collected data prospectively, enrolled a diverse group of patients from multiple centers, and adjusted for a large number of factors associated with severity of illness, medication use, and treatment outcome. In addition to confirming the benefits of ART, we also found an independent survival benefit to using fluconazole prophylaxis during TB treatment, likely because fungal opportunistic infections are a major problem in Southeast Asia [26]. Because over 80% of patients in this study met immunological criteria for AIDS, we expected that co-trimoxazole, which is internationally recommended as prophylaxis against *Pneumocystis jirovecii *pneumonia, toxoplasmosis, and other infections, would increase survival, but the point estimate was only statistically significant in the all patients analysis, not the subset of bacteriologic-confirmed patients [27]. In Africa, some of co-trimoxazole's benefit has been ascribed to reducing malaria deaths [28]. It is possible that the magnitude of benefit from co-trimoxazole is not as great among HIV-infected TB patients that are receiving ART and living in settings with less malaria, such as Thailand. It is also possible that patients did not initiate co-trimoxazole early enough to reduce HIV-associated deaths that occurred early during TB treatment, that antimicrobial resistance mitigated the benefit of co-trimoxazole, or that our study was under-powered to show a strong beneficial effect. Further prospective studies would help define the added value of co-trimoxazole and fluconazole to ART in reducing mortality during TB treatment.

International guidelines recommend that HIV-infected persons initiate treatment with nevirapine, zidovudine, and lamivudine; because rifampin can alter drug levels of nevirapine, guidelines recommend that efavirenz replace nevirapine in patients receiving rifampin [29]. The high cost of switching from nevirapine to efavirenz has led some physicians in resource-limited settings to continue using nevirapine in HIV-infected TB patients, a decision backed by a pharmacokinetic study in Thailand that showed good HIV and TB treatment outcomes in patients receiving nevirapine and rifampin at the same time [30]. Our study is unique, because it compared the frequency of adverse events and TB treatment outcomes in patients receiving either efavirenz or nevirapine containing regimens during TB treatment. In our study, adverse events and successful TB outcome occurred with similar frequency in both groups. Although nevirapine is frequently associated with rash, we found that rash was infrequent in both ART groups; in fact, patients not treated with ART reported rash more frequently, likely because of progressive cutaneous disease associated with declining CD4 count [31]. We did not, however, serially evaluate HIV RNA viral load or CD4 to assess the benefits of nevirapine vs. efavirenz in achieving HIV treatment success. Current international guidelines also recommend that patients with advanced immune-suppression begin ART within two weeks to two months of beginning TB treatment [2]. Although our study supports this recommendation, we were only able to demonstrate statistical significance for ART initiated within four months of TB treatment. The non-randomised design, relatively small sample size, lack of pharmacokinetic data, and lack of detailed efficacy and safety endpoints make our data about choice of NNRTI and timing of ART initiation hypothesis generating. Studies with larger sample sizes, controlled designs, and more extensive clinical monitoring data are needed to further assess the appropriateness of using nevirapine and rifampin concomitantly and to define the best time to initiate ART.

Our study is subject to important limitations. First, even though our study was derived from a diverse population base, some patients refused HIV testing, and our analysis was limited to a subset of HIV-infected TB patients, due to strict eligibility criteria and specific research questions. It is possible that patients included in our analysis differ in important ways from patients not included, such as those previously treated for TB, and that separate studies of these populations are needed. Second, patients only underwent three study visits, even though treatment was for a minimum of six months, and patients had limited laboratory monitoring. It is possible, therefore, that ascertainment of adverse events, e.g., hepatitis, was incomplete. Third, because this was an observational study, patients who developed adverse events underwent evaluation by their individual physicians. We do not have sufficient information to assign a specific toxicity grade to each adverse event.

## Conclusion

In conclusion, our study highlights important issues about the timing and choice of ART and the added benefit of opportunistic infection prophylaxis in patients receiving ART and TB treatment. Unfortunately, ART remains underused in HIV-infected TB patients in Thailand and other resource-limited settings. Reducing mortality in this population will require intensive collaboration between HIV and TB programs to insure prompt HIV testing and initiation of life-saving treatment.

## Competing interests

The authors declare that they have no competing interests.

## Authors' contributions

JKV, SN, SA, WM, CS, WS, CB, PM, CDW, and JWT designed the study. SN, SA, WM, CS, WS, CB, WK, PM, and KPC collected data. JKV, KPC, CB, WK, and PM analyzed data. JKV and WK drafted the manuscript. All provided critical comments for revision and approved the final version of the manuscript.

## Pre-publication history

The pre-publication history for this paper can be accessed here:

http://www.biomedcentral.com/1471-2334/9/42/prepub

## Supplementary Material

Additional file 1 **Tables 1–5**. Table 1. Characteristics of HIV-infected tuberculosis patients, stratified by vital status at the end of tuberculosis treatment. Table 2. Mortality rate among HIV-infected TB patients, stratified by microbiologic status. Table 3. Multivariable Cox proportional hazards analysis of risk factors for death among HIV-infected TB patients, stratified by microbiologic status. Table 4. Adverse events in all HIV-infected tuberculosis patients, including patients not treated with anti-retroviral therapy (ART), patients treated with nevirapine-containing ART, and patients treated with efavirenz-containing ART. Table 5. Multivariable Cox proportional hazards analysis of risk factors for death or death and default among HIV-infected tuberculosis patients, stratified by HIV anti-retroviral therapy regimen. Click here for file
