# Supplementary material for: HIV care and treatment factors associated with improved survival during TB treatment in Thailand: an observational study
Source: BMC Infect Dis. 2009 Apr 13;9:42. doi: 10.1186/1471-2334-9-42 (PMC2674442; doi:10.1186/1471-2334-9-42)
Supplement: Additional file 1 — Tables 1–5. Table 1. Characteristics of HIV-infected tuberculosis patients, stratified by vital status at the end of tuberculosis treatment. Table 2. Mortality rate among HIV-infected TB patients, stratified by microbiologic status. Table 3. Multivariable Cox proportional hazards analysis of risk factors for death among HIV-infected TB patients, stratified by microbiologic status. Table 4. Adverse events in all HIV-infected tuberculosis patients, including patients not treated with anti-retroviral therapy (ART), patients treated with nevirapine-containing ART, and patients treated with efavirenz-containing ART. Table 5. Multivariable Cox proportional hazards analysis of risk factors for death or death and default among HIV-infected tuberculosis patients, stratified by HIV anti-retroviral therapy regimen. [file 1471-2334-9-42-S1.doc]

**Additional Files, Table 1. Characteristics of HIV-infected tuberculosis patients, stratified by vital status at the end of tuberculosis treatment.**

| Characteristics and clinical features | All patients | | Survived | | Died^††^ | | p |
| --- | --- | --- | --- | --- | --- | --- | --- |
|  | (n=667) | | (n=555) | | (n=112) | |  |
|  | N | % | n | % | n | % |  |
| **Characteristics** |  |  |  |  |  |  |  |
| Age >34 years | 332 | 49.8 | 281 | 50.1 | 51 | 45.5 | 0.32 |
| Male | 461 | 69.1 | 383 | 69.0 | 78 | 69.6 | 0.89 |
| Disease classification |  |  |  |  |  |  |  |
| Pulmonary TB | 387 | 58.0 | 330 | 59.5 | 57 | 50.1 | 0.24 |
| Smear positive (n=363)^*^ | 234 | 64.5 | 199 | 60.3 | 35 | 61.4 |  |
| Extra-pulmonary TB | 206 | 30.9 | 166 | 29.9 | 40 | 35.7 |  |
| Both | 74 | 11.1 | 59 | 10.6 | 15 | 13.4 |  |
| Hospitalized at enrollment | 189 | 28.3 | 134 | 24.1 | 55 | 49.1 | <0.01 |
| DOT by healthcare worker/village health volunteer | 191 | 28.6 | 159 | 28.6 | 32 | 28.6 | 0.99 |
| Received standard or extended HRZE regimen | 610 | 91.5 | 510 | 91.9 | 100 | 89.3 | 0.33 |
| History of injection drug use | 161 | 24.1 | 138 | 24.9 | 23 | 20.5 | 0.37 |
| **History of present illness** |  |  |  |  |  |  |  |
| Cough | 489 | 73.3 | 404 | 72.8 | 85 | 75.9 | 0.50 |
| Cough more than 2 weeks | 285 | 58.3 | 246 | 60.9 | 39 | 45.9 | 0.01 |
| Fever | 493 | 73.9 | 418 | 75.3 | 75 | 67.0 | 0.06 |
| Diarrhea | 172 | 25.8 | 135 | 24.3 | 37 | 33.0 | 0.06 |
| Self-reported body weight loss | 545 | 81.7 | 446 | 80.4 | 99 | 88.4 | 0.03 |
| Hemoptysis | 66 | 9.9 | 57 | 10.3 | 9 | 8.0 | 0.47 |
| Difficulty breathing | 284 | 42.6 | 229 | 41.3 | 55 | 49.1 | 0.13 |
| Delay in HIV diagnosis (n=442)^*, †^ | 327 | 74.0 | 271 | 72.8 | 56 | 80.0 | 0.21 |
| Delay in seeking TB care^§^ | 324 | 48.6 | 269 | 48.5 | 55 | 49.1 | 0.90 |
| Severe TB disease | 292 | 43.8 | 236 | 42.5 | 56 | 50.0 | 0.15 |
| **Medicines taken during TB treatment**^¶^ |  |  |  |  |  |  |  |
| Received TB regimen of unknown efficacy or that was likely to be ineffective due to drug resistance | 67 | 10.0 | 53 | 9.5 | 14 | 12.5 | 0.34 |
| Co-trimoxazole | 553 | 82.9 | 474 | 85.4 | 79 | 70.5 | <0.01 |
| Fluconazole | 376 | 56.4 | 325 | 58.6 | 51 | 45.6 | 0.01 |
| Anti-retroviral drugs | 273 | 40.9 | 249 | 44.9 | 24 | 21.4 | <0.01 |
| **Laboratory studies** |  |  |  |  |  |  |  |
| Diagnosed with multi-drug resistant TB(n=654)^*^ | 16 | 2.4 | 11 | 2.0 | 5 | 4.5 | 0.12 |
| Diagnosed with HIV before TB diagnosis | 440 | 66.0 | 363 | 65.4 | 77 | 68.7 | 0.50 |
| CD4 count at enrollment <200 cells/uL (n=653)^*^ | 535 | 81.9 | 438 | 80.4 | 97 | 89.8 | 0.20 |
| CD4 (median, IQR) (n=653)^*^ | 60, 23-146 |  | 65, 28-166 |  | 30, 11-93 |  |  |
| HIV viral load at enrollment ≥100,000 copies/mL (n=78)^*^ | 52 | 66.7 | 46 | 66.7 | 6 | 66.7 | 1.00 |
| HIV viral load at enrollment (median*1000, IQR) (n=78)^*^ | 313, 52-785 |  | 319, 68-759 |  | 228, 30-785 |  |  |
| HBsAg reactive (n=651)^*^ | 54 | 8.3 | 46 | 8.4 | 8 | 7.8 | 0.83 |
| Anti-HCV reactive (n=651)^*^ | 192 | 29.5 | 155 | 28.3 | 37 | 35.9 | 0.12 |
| HBsAg and anti-HCV reactive (n=651)^*^ | 19 | 2.9 | 14 | 2.6 | 5 | 4.9 | 0.20 |
| Abnormal chest x-ray (n=597)^*^ | 471 | 78.9 | 394 | 79.1 | 77 | 77.8 | 0.77 |
| Abnormal liver enzyme levels^**^ | 110 | 16.5 | 78 | 14.2 | 32 | 29.4 | <0.01 |
| Hemoglobin <10 g/dL (n=662)^*^ | 324 | 48.9 | 261 | 47.3 | 63 | 57.3 | 0.06 |
| Platelet >150,000 cells/uL (n=662)^*^ | 75 | 11.3 | 56 | 10.1 | 19 | 17.3 | 0.03 |
| Creatinine ≥1.5 mg/dL (n=661)^*^ | 30 | 4.5 | 22 | 4.0 | 8 | 7.3 | 0.12 |
| Albumin ≤2.5 g/dL (n=656)^*^ | 148 | 22.6 | 106 | 19.3 | 42 | 39.2 | <0.01 |
| **Physical examination** |  |  |  |  |  |  |  |
| Body mass index <18.5 kg/m^2^ | 383 | 57.4 | 309 | 55.7 | 74 | 66.1 | 0.04 |
| Bacille Calmette-Guérin scar present | 441 | 66.1 | 376 | 67.7 | 65 | 58.0 | 0.04 |
| **Treatment outcomes** |  |  |  |  |  |  |  |
| Cure/completed | 430 | 64.5 | 430 | 77.5 | 0 | 0 |  |
| Failed | 4 | 0.6 | 4 | 0.7 | 0 | 0 |  |
| Defaulted | 55 | 8.2 | 55 | 9.9 | 0 | 0 |  |
| Died | 112 | 16.8 | 0 | 0 | 112 | 100.0 |  |
| Transferred out | 66 | 9.9 | 66 | 11.9 | 0 | 0 |  |

TB, tuberculosis; HIV, human immunodeficiency virus; DOT, directly observed therapy; HRZE, isoniazid, rifampin, pyrazinamide, ethambutol; HBsAg, hepatitis B surface antigen; HCV, hepatitis C virus

^*^Among those with available results

^†^CD4 count at time of HIV diagnosis <200 cells/μL

^§^Reported coughing before TB diagnosis lasting >1 month or had severe symptoms lasting >14 days and self-assessed intensity score >5

^¶^Those who started medicines (co-trimoxazole, fluconazole, and anti-retroviral therapy) <30 days before end of TB treatment were classified as not receiving medicines during TB treatment

^**^Aspartate aminotransperase >120 mEq/L, alanine aminotransferase >165 mEq/L, or total bilirubin >2 mg/dL

^††^Those who died within 90 days of default were classified as having died during TB treatment

**Additional Files, Table 2. Mortality rate among HIV-infected TB patients, stratified by microbiologic status.**

| Risk factors | All TB patients  (n=337) | | | Bacteriologic-confirmed TB patients (n=232) | | |
| --- | --- | --- | --- | --- | --- | --- |
|  | Rate^††^ | 95% CI | | Rate^††^ | 95% CI | |
|  |  | Lower | Upper |  | Lower | Upper |
| Delay in HIV diagnosis^*^ |  |  |  |  |  |  |
| Yes | 31.0 | 23.8 | 40.2 | 26.7 | 19.1 | 37.4 |
| No | 21.0 | 12.4 | 35.5 | 24.4 | 13.5 | 44.1 |
| Unknown | 35.1 | 26.0 | 47.5 | 30.0 | 19.9 | 45.2 |
| Delay in seeking TB care^†^ |  |  |  |  |  |  |
| Yes | 31.3 | 24.0 | 40.7 | 28.6 | 20.8 | 39.2 |
| No | 29.8 | 23.0 | 38.7 | 25.9 | 18.1 | 37.0 |
| Hospitalized at enrollment |  |  |  |  |  |  |
| Yes | 55.5 | 42.6 | 72.2 | 59.1 | 43.5 | 80.3 |
| No | 21.3 | 16.4 | 27.6 | 15.0 | 10.3 | 21.9 |
| Severe TB disease^§^ |  |  |  |  |  |  |
| Yes | 34.4 | 26.5 | 44.7 | 31.1 | 21.7 | 44.4 |
| No | 27.4 | 21.1 | 35.6 | 24.9 | 18.1 | 34.3 |
| CD4 at enrollment <200 cells/µL |  |  |  |  |  |  |
| Yes | 33.3 | 27.3 | 40.6 | 29.7 | 23.0 | 38.3 |
| No | 15.6 | 8.6 | 28.2 | 17.5 | 8.7 | 34.9 |
| Took co-trimoxazole during TB treatment^**^ |  |  |  |  |  |  |
| Yes | 24.9 | 20.0 | 31.1 | 20.4 | 15.2 | 27.3 |
| No | 65.7 | 46.4 | 92.9 | 81.0 | 53.8 | 121.9 |
| Took fluconazole during TB treatment^¶^ |  |  |  |  |  |  |
| Yes | 21.8 | 16.6 | 28.7 | 17.5 | 12.0 | 25.5 |
| No | 45.8 | 35.6 | 59.0 | 44.0 | 32.4 | 59.7 |
| Took ART during TB treatment^**^ |  |  |  |  |  |  |
| Yes | 13.1 | 8.8 | 19.6 | 8.7 | 4.8 | 15.7 |
| No | 47.8 | 38.8 | 58.9 | 46.4 | 35.8 | 60.2 |
| Received TB regimen of unknown efficacy or that was likely to be ineffective due to drug resistance |  |  |  |  |  |  |
| Yes | 35.0 | 20.7 | 59.1 | 44.7 | 24.7 | 80.7 |
| No | 30.0 | 24.6 | 36.5 | 25.4 | 19.6 | 32.9 |
| Hemoglobin <10 g/dL |  |  |  |  |  |  |
| Yes | 35.7 | 27.9 | 45.7 | 32.2 | 23.7 | 43.7 |
| No | 25.0 | 18.7 | 33.2 | 21.0 | 14.2 | 31.0 |
| Platelet >150,000 cells/µL |  |  |  |  |  |  |
| Yes | 48.8 | 31.2 | 76.6 | 49.9 | 28.3 | 87.9 |
| No | 28.0 | 22.8 | 34.3 | 24.2 | 18.5 | 31.6 |
| Creatinine ≥1.5 mg/dL |  |  |  |  |  |  |
| Yes | 62.2 | 31.1 | 124.3 | 65.4 | 27.2 | 157.2 |
| No | 28.7 | 23.6 | 34.9 | 25.5 | 19.9 | 32.8 |
| Albumin ≤2.5 g/dL |  |  |  |  |  |  |
| Yes | 56.6 | 41.9 | 76.6 | 56.8 | 40.0 | 80.8 |
| No | 22.6 | 17.7 | 28.8 | 17.8 | 12.7 | 25.0 |
| Unknown | 95.6 | 39.8 | 229.7 | 80.4 | 25.9 | 249.3 |
| Abnormal liver enzyme level^**^ |  |  |  |  |  |  |
| Yes | 58.0 | 41.0 | 82.0 | 48.7 | 31.7 | 74.6 |
| No | 25.1 | 20.1 | 31.3 | 22.7 | 17.0 | 30.3 |
| HBsAg reactive |  |  |  |  |  |  |
| Yes | 28.4 | 14.2 | 56.7 | 21.9 | 8.2 | 58.2 |
| No | 28.4 | 23.3 | 34.8 | 24.6 | 18.9 | 31.9 |
| Unknown | 185.6 | 96.6 | 356.7 | 284.6 | 142.3 | 569.1 |
| Anti-HCV reactive |  |  |  |  |  |  |
| Yes | 37.5 | 27.2 | 51.8 | 35.2 | 23.4 | 53.0 |
| No | 25.0 | 19.7 | 31.9 | 20.5 | 14.8 | 28.2 |
| DOT by healthcare worker/village health volunteer |  |  |  |  |  |  |
| Yes | 30.9 | 21.8 | 43.7 | 25.5 | 16.3 | 40.0 |
| No | 30.4 | 24.4 | 37.8 | 28.1 | 21.2 | 37.2 |

TB, tuberculosis; HIV, human immunodeficiency virus; HR, hazard ratio; CI, confidence interval; HBsAg, hepatitis B surface antigen; HCV, hepatitis C virus; DOT, directly observed therapy

^*^CD4 count at time of HIV diagnosis <200 cells/μL

^†^Reported coughing before TB diagnosis lasting >1 month or had severe symptoms lasting >14 days and self-assessed intensity score >5

^§^Patients with disseminated, meningeal, abdominal, intestinal TB or other forms of extra-pulmonary TB (except for peripheral lymphatic TB) or those with all of the following characteristics: self-reported weight loss, coughing up blood, having difficulty breathing in past 4 weeks before TB diagnosis, and having cavitary TB or >1/3 involvement of either lung at the initial evaluation

^¶^Those who started medicines <30 days before end of TB treatment were classified as not receiving medicines during TB treatment

^**^Aspartate transaminase ≥120 mEq/L, alanine aminotransferase ≥165 mEq/L or total bilirubin >2 mg/dL

^††^Rate per 100 person-year

**Additional Files, Table 3. Multivariable Cox proportional hazards analysis of risk factors for death among HIV-infected TB patients, stratified by microbiologic status.**

| Risk factors | All TB patients  (n=337) | | | Bacteriologic-confirmed TB patients (n=232) | | |
| --- | --- | --- | --- | --- | --- | --- |
|  | HR | 95% CI | | HR | 95% CI | |
|  |  | Lower | Upper |  | Lower | Upper |
| Delay in HIV diagnosis^*^ |  |  |  |  |  |  |
| Yes | 2.41 | 0.81 | 7.19 | 1.71 | 0.36 | 8.12 |
| No | Ref |  |  | Ref |  |  |
| Unknown | **4.27** | **1.42** | **12.84** | 2.61 | 0.59 | 11.43 |
| Delay in seeking TB care^†^ |  |  |  |  |  |  |
| Yes | 0.56 | 0.31 | 1.00 | 0.51 | 0.25 | 1.04 |
| No | Ref |  |  | Ref |  |  |
| Hospitalized at enrollment |  |  |  |  |  |  |
| Yes | **2.00** | **1.07** | **3.75** | **3.10** | **1.35** | **7.12** |
| No | Ref |  |  | Ref |  |  |
| Severe TB disease^§^ |  |  |  |  |  |  |
| Yes | 0.99 | 0.54 | 1.79 | 1.09 | 0.45 | 2.63 |
| No | Ref |  |  | Ref |  |  |
| CD4 at enrollment^¶^ | **0.99** | **0.99** | **1.00** | **0.99** | **0.98** | **1.00** |
| Took co-trimoxazole during TB treatment^**^ |  |  |  |  |  |  |
| Yes | **0.41** | **0.20** | **0.83** | 0.38 | 0.14 | 1.03 |
| No | Ref |  |  | Ref |  |  |
| Took fluconazole during TB treatment^**^ |  |  |  |  |  |  |
| Yes | **0.34** | **0.18** | **0.64** | **0.27** | **0.12** | **0.62** |
| No | Ref |  |  | Ref |  |  |
| Took ART during TB treatment^**^ |  |  |  |  |  |  |
| Yes | **0.16** | **0.07** | **0.36** | **0.06** | **0.02** | **0.23** |
| No | Ref |  |  | Ref |  |  |
| Received TB regimen of unknown efficacy or that was likely to be ineffective due to drug resistance |  |  |  |  |  |  |
| Yes | 1.98 | 0.93 | 4.23 | 2.18 | 0.73 | 6.54 |
| No | Ref |  |  | Ref |  |  |
| Hemoglobin <10 g/dL |  |  |  |  |  |  |
| Yes | 0.95 | 0.51 | 1.76 | 0.34 | 0.14 | 0.83 |
| No | Ref |  |  | Ref |  |  |
| Platelet >150,000 cells/µL |  |  |  |  |  |  |
| Yes | 1.01 | 0.50 | 2.03 | 1.00 | 0.39 | 2.54 |
| No | Ref |  |  | Ref |  |  |
| Creatinine ≥1.5 mg/dL |  |  |  |  |  |  |
| Yes | 1.34 | 0.41 | 4.38 | 3.59 | 0.42 | 30.78 |
| No | Ref |  |  | Ref |  |  |
| Albumin ≤2.5 g/dL |  |  |  |  |  |  |
| Yes | 1.75 | 0.89 | 3.44 | 2.18 | 0.82 | 5.80 |
| No | Ref |  |  | Ref |  |  |
| Unknown | **21.06** | **4.08** | **108.55** | **55.24** | **8.35** | **365.45** |
| Abnormal liver enzyme level^††^ |  |  |  |  |  |  |
| Yes | 1.46 | 0.73 | 2.91 | 1.68 | 0.69 | 4.08 |
| No | Ref |  |  | Ref |  |  |
| HBsAg reactive |  |  |  |  |  |  |
| Yes | 1.23 | 0.45 | 3.36 | 1.12 | 0.23 | 5.42 |
| No | Ref |  |  | Ref |  |  |
| Unknown | 4.03 | 0.78 | 20.83 | 4.61 | 0.55 | 38.67 |
| Anti-HCV reactive |  |  |  |  |  |  |
| Yes | 1.34 | 0.75 | 2.38 | 1.42 | 0.62 | 3.24 |
| No | Ref |  |  | Ref |  |  |
| DOT by healthcare worker/village health volunteer |  |  |  |  |  |  |
| Yes | 0.66 | 0.36 | 1.19 | **0.30** | **0.13** | **0.74** |
| No | Ref |  |  | Ref |  |  |

TB, tuberculosis; HIV, human immunodeficiency virus; HR, hazard ratio; CI, confidence interval; HBsAg, hepatitis B surface antigen; HCV, hepatitis C virus; DOT, directly observed therapy; Ref, reference; potential confounders and variables for which p≤0.20 in bivariate analyses were included in multivariate Cox proportional hazard analyses; those who started co-trimoxazole, fluconazole, and anti-retroviral therapy before TB diagnosis were excluded; bold type indicates statistical significance (p<0.05)

^*^CD4 count at time of HIV diagnosis <200 cells/μL

^†^Reported coughing before TB diagnosis lasting >1 month or had severe symptoms lasting >14 days and self-assessed intensity score >5

^§^Patients with disseminated, meningeal, abdominal, intestinal TB or other forms of extra-pulmonary TB (except for peripheral lymphatic TB) or those with all of the following characteristics: self-reported weight loss, coughing up blood, having difficulty breathing in past 4 weeks before TB diagnosis, and having cavitary TB or >1/3 involvement of either lung at the initial evaluation

^¶^Continuous variable

^**^Those who started medicines <30 days before end of TB treatment were classified as not receiving medicines during TB treatment

^††^Aspartate transaminase ≥120 mEq/L, alanine aminotransferase ≥165 mEq/L or total bilirubin >2 mg/dL

**Additional Files, Table 4. Adverse events in all HIV-infected tuberculosis patients, including patients not treated with anti-retroviral therapy (ART), patients treated with nevirapine-containing ART, and patients treated with efavirenz-containing ART.**

| Adverse events | All patients (n=653) | | ART-untreated (n=393) | | Received ART regimen containing | | | |
| --- | --- | --- | --- | --- | --- | --- | --- | --- |
|  |  |  |  |  | NVP (n=147) | | EFV (n=113) | |
|  | n | % | n | % | n | % | n | % |
| Rash | 103 | 15.8 | 72 | 18.3 | 18 | 12.2 | 13 | 11.5 |
| Liver disease | 37 | 5.7 | 19 | 4.8 | 8 | 5.4 | 10 | 8.8 |
| Diarrhea | 33 | 5.0 | 25 | 6.4 | 2 | 1.4 | 6 | 5.3 |
| Pneumonia, including *Pneumocystis jirovecii* pneumonia | 25 | 3.8 | 18 | 4.6 | 3 | 2.0 | 4 | 3.5 |
| Immune reconstitution infammatory syndrome^*^ | 14 | 2.1 | n/a | n/a | 7 | 4.8 | 7 | 6.2 |
| Death | 111 | 17.0 | 88 | 22.4 | 12 | 8.2 | 11 | 9.7 |

TB, tuberculosis; HIV, human immunodeficiency virus; ART, anti-retroviral therapy; NVP, nevirapine; EFV, efavirenz; n/a, not applicable; those who received unspecified ART regimens (n=4), ART regimens other than NVP and EFV (n=3), and both NVP and EFV regimens with no date/reason of regimen change documented (n=6) were excluded

^*^Among those eligible to be evaluated

**Additional Files, Table 5. Multivariable Cox proportional hazards analysis of risk factors for death or death and default among HIV-infected tuberculosis patients, stratified by HIV anti-retroviral therapy regimen.**

| *TB patients with cure, complete, death, and default outcomes* | | | | | | | | |
| --- | --- | --- | --- | --- | --- | --- | --- | --- |
| Comparison groups | | | All patients  (n=432) | | | Bacteriologic-confirmed TB (n=301) | | |
|  |  |  | HR | 95% CI | | HR | 95% CI | |
|  |  |  |  | Lower | Upper |  | Lower | Upper |
| Nevirapine | vs. | no ART | **0.09** | **0.03** | **0.30** | **0.10** | **0.03** | **0.36** |
| Efavirenz | vs. | no ART | **0.33** | **0.13** | **0.83** | **0.05** | **0.01** | **0.44** |
| Nevirapine | vs. | Efavirenz | 0.26 | 0.06 | 1.01 | 1.86 | 0.17 | 19.86 |
|  |  |  |  |  |  |  |  |  |
| *TB patients with cure, complete, and death outcomes only* | | | | | |  |  |  |
| Comparison groups | | | All patients  (n=397) | | | Bacteriological-confirmed TB (n=272) | | |
|  |  |  | HR | 95% CI | | HR | 95% CI | |
|  |  |  |  | Lower | Upper |  | Lower | Upper |
| Nevirapine | vs. | no ART | **0.08** | **0.02** | **0.28** | **0.09** | **0.03** | **0.33** |
| Efavirenz | vs. | no ART | **0.32** | **0.13** | **0.83** | **0.05** | **0.01** | **0.34** |
| Nevirapine | vs. | Efavirenz | 0.26 | 0.06 | 1.11 | 2.03 | 0.19 | 21.69 |

TB, tuberculosis, HIV, human immunodeficiency virus; HR, hazard ratio; CI, confidence interval; ART, anti-retroviral therapy; bold type indicates statistical significance (p<0.05)

Hazard ratios from multivariate Cox proportional hazard analyses adjusted for delay of HIV diagnosis and seeking TB care, CD4, TB disease severity, co-trimoxazole use, fluconazole use, laboratory findings, TB regimens, and whether or not hospitalized at enrollment, received directly observed therapy, and been treated for TB previously
